# Clinician perspectives on antithrombotic therapy management in advanced cancer: a multinational qualitative study

**DOI:** 10.1016/j.rpth.2026.103427

**Published:** 2026-03-25

**Authors:** Elin Baddeley, Carme Font, Isabelle Mahé, Michelle Edwards, Stephanie Sivell, Kate J. Lifford, Victoria Mailen Arfuch, Nuri Coma-Auli, Mette Søgaard, Helle Enggaard, Hélène Helfer, Nassima Si Mohammed, Kathy Seddon, Simon P. Mooijaart, Mark Pearson, Sebastian Szmit, F.A. Klok, Simon Noble, Anette Arbjerg Højen, SERENITY consortium

**Affiliations:** 1 Marie Curie Research Centre (MCRC), Division of Population Medicine, School of Medicine, Cardiff University, Cardiff, UK; 2 Department of Medical Oncology, Hospital Clinic Barcelona, Barcelona, Spain; 3 Paris Cité Université, APHP, Louis Mourier Hospital, Internal Medicine Department, Inserm UMR-S970, Paris Cardiovascular Research Center, Team “Endotheliopathy and Hemostasis Disorders,” Paris, France; 4 Wales Centre for Primary and Emergency Care Research (PRIME Centre Wales), Division of Population Medicine, School of Medicine, Cardiff University, Cardiff, UK; 5 Division of Clinical Neuroscience, Department of Medical Sciences, Uppsala University, Uppsala, Sweden; 6 Danish Center for Health Services Research, Aalborg University Hospital, Aalborg, Denmark; 7 Center for General Practice, Aalborg University, Aalborg, Denmark; 8 Clinical Nursing Research Unit & Clinical Cancer Research Center, Aalborg University Hospital, Aalborg, Denmark; 9 Internal Medicine Department, Clinical Research Unit, Assistance Publique-Hôpitaux de Paris, Hôpital Bichat, Paris, France; 10 Department of Internal Medicine, Leiden University Medical Center (LUMC), Leiden, Netherlands; 11 LUMC Center for Medicine for Older People, Faculty of Medicine, Leiden University Medical Center, Leiden, Netherlands; 12 Wolfson Palliative Care Research Centre, Hull York Medical School, University of Hull, Hull, UK; 13 Department of Cardio-Oncology, Centre of Postgraduate Medical Education, Warsaw, Poland; 14 Department of Medicine—Thrombosis and Hemostasis, Leiden University Medical Center, Leiden, Netherlands

**Keywords:** antithrombotic therapy, cancer, end of life, qualitative research, palliative care

## Abstract

**Background:**

Decision making about antithrombotic therapy (ATT) in patients with advanced cancer near the end of life is fraught with clinical uncertainty and can significantly affect care. Despite its importance and complexity, ATT is often deprioritized or guided by legacy prescribing and monitoring patterns. Management spans multiple specialties, with roles and responsibilities frequently blurred. Clinicians’ perspectives remain largely underexplored, which are crucial to inform improved care models.

**Objectives:**

This study explores clinicians’ experiences of current practice of continuing and deprescribing ATT in patients with advanced cancer at the end of life.

**Methods:**

Qualitative methodology using semistructured interviews with clinicians involved in ATT management at the end of life, across Denmark, France, Spain, and the United Kingdom. Data were analyzed using Framework Analysis.

**Results:**

Eighty clinicians across a range of specialties were interviewed. Two major themes were generated: (1) balancing complexities in ATT management: clinicians reported several challenges, from ambiguity surrounding roles and responsibilities, delicacy around timing, and variance in risk perceptions of ATT, balanced with patient preferences; and (2) culture of continuation: clinicians described a general and ATT-specific culture of continuation and reported a passivity in relation to ATT review.

**Conclusion:**

The management of ATT in this context is multifaceted, influenced by many competing factors. These complexities need to be understood and addressed to support decision making related to ATT at the end of life.

## Essentials

• Clinician perspectives on antithrombotic therapy (ATT) in advanced cancer are underexplored.
• Clinicians across 4 countries and specialties involved in ATT management were interviewed.
• Evidence, guidance, and support for ATT decision making at the end of life are limited.
• ATT is often among the last medications stopped, reflecting a culture of continuation.

## 1 Introduction

Advance care planning (ACP) for patients with terminal and life-limiting illnesses has become standard practice in many European countries. Deprescribing, a core component of ACP, is particularly relevant toward the end of life when altered pharmacokinetics and pharmacodynamics can increase susceptibility to adverse events [1]. Cancer accounts for over one-quarter (25.8%) of deaths across Europe. Among these patients, 5% to 15% receive anticoagulants for indications including venous thromboembolism, or stroke prevention in atrial fibrillation or mechanical heart valves, while 25% to 35% receive antiplatelet agents for the management of arterial disease, including ischemic heart and peripheral vascular disease [[2], [3], [4]]. Recent evidence suggests that most patients with cancer who are prescribed antithrombotic therapy (ATT), including anticoagulants and antiplatelet agents, continue treatment until death [[5], [6], [7], [8]]. This is of concern since the association between continued ATT use and increased bleeding risk is well recognized and associated with reduced quality of life [[9], [10], [11]], and bleeding events, even minor bleeds, can have a significant impact on patients, their carers, and health care professionals [12,13].

The decision to deprescribe ATT at the end of life in patients with advanced cancer is complex and characterized by considerable clinical uncertainty. Current guidelines offer little direction regarding long-term ATT management at the end of life [[14], [15], [16], [17]]. The challenges of deprescribing medications within the context of ACP are well documented but usually relate to the principle of deprescribing as a whole, rather than cessation of specific medications [18]. In clinical practice, this results in perceived nonessential medications being deprescribed first, and sidestepping more contentious medications such as anticoagulants, which have a greater risk of harm [19]. Similarly, while clinician perspectives on deprescribing in general have been captured, there are a paucity of data regarding their perspectives on ATT in patients with advanced cancer at the end of life. However, as ATT is indicated for a myriad of conditions, it could be initiated by many different medical specialists; therefore, a variety of perspectives need to be captured in order to fully understand current practices and challenges. Additionally, there is a growing body of opinion, which supports the need to develop interventions to facilitate deprescribing for patients accessing palliative care services [20].

This qualitative study forms a component of SERENITY, a multimethod, pan-European project funded by the European Union, aimed to develop and evaluate a shared decision-support tool (SDST) to reconsider treatment decisions regarding ATT in cancer patients at the end of life [21]. These interviews, alongside a realist review [22], clinician survey [6], epidemiological data [5,7,8] patient interviews [23], and Delphi-consensus survey [24], will inform the development and implementation of the SDST, which will be evaluated in a randomized controlled trial. The aim of this qualitative study was to explore clinicians’ experiences of current practice of continuing and deprescribing ATT, in patients with cancer at the end of life.

## 2 Methods

### 2.1 Study design

Qualitative study based on semistructured interviews with clinicians involved in ATT management, across Denmark (DK), France (FR), Spain (SP), and the United Kingdom (UK), chosen to represent a large part of the European Union, in addition to the research teams’ experience in related research. This study was underpinned by a phenomenological hermeneutics approach. This study is reported according to the Consolidated Criteria for Reporting Qualitative research [25].

### 2.2 Setting and recruitment

Clinicians were purposively sampled across a variety of specialties involved in ATT management at end of life. Participants were recruited across public and private health services, hospitals, tertiary care centers, and specialized care units, in DK, FR, SP, and the UK, between April 2023 and July 2024. Sampling across these European countries was undertaken to capture a breadth of experiences and perspectives and informed by previous research, highlighting that lead clinicians responsible for initiation and continuation of ATT in cancer patients may vary between and within countries [26].

Clinicians were invited to participate by email, identified through their hospitals’ management and directories, based on prespecified inclusion criteria, including at least 10 years of practice in their specialty to ensure sufficient expertise. It was anticipated that 72 to 96 clinicians would need to be recruited, to allow for adjustments in sampling as needed. Recruitment strategies aimed to capture a fair representation of specialties involved in ATT management, both across and within countries, while still allowing flexibility in recruitment between countries as relevant. For example, a decision was later made to recruit palliative care nurse specialists as their role in ATT management in DK, SP, and the UK became evident. In FR, this role was less clearly reflected during the recruitment process, and palliative care nurses were therefore not recruited.

### 2.3 Data collection

Semistructured interviews were conducted in person or via videoconferencing. The research teams collaborated on the development of the interview guide, initially developed in English, tailored to the research aim, and informed by a realist review conducted as part of SERENITY [22], the latter of which also informed the definition used to clarify the end-of-life phase for the purpose of the study. While there is currently no consensus in the literature regarding the term end-of-life, as part of the review, the following definition was developed: “The last phase of life is estimated to be within 12 months before dying, in which a person is living with an eventually fatal condition, even if the prognosis is ambiguous or unknown. End of life is the phase of life roughly 3 months before dying in which a person is living with a fatal condition in which the death prognosis is fairly certain…” [22]. During interviews, the surprise question “would you be surprised if this patient died within the next year” was incorporated into the interview guide to prompt and explore how clinicians defined what they would consider end of life in the context of this population [27].

Once finalized, the interview guide was translated into the native language as relevant. Participant demographic information was collected alongside interviews. Data on those who refused to participate were not collected; no participants dropped out during the study. Each research team was responsible for local data collection (UK: E.B., M.E., K.L., S.S., and S.N.; DK: A.A.H., M.S., and H.E.; SP: C.F., V.A., and N.C.-A.; FR: I.M., H.H., N.S.M., and medical students). Each of the research teams comprised relevant clinical and/or qualitative expertise, particularly in the field of thrombosis research in the context of cancer. Regular meetings were held throughout data collection to ensure a common and consistent approach across all research teams. Interviews were audio recorded and transcribed verbatim by a member of the research team (DK, FR, and SP) or agency (the UK) and checked by a second researcher. Participants were offered the opportunity to review their transcript.

### 2.4 Data analysis

Data were analyzed using Framework Analysis, a qualitative method with 5 interconnected steps: familiarization, thematic framework, indexing, charting, mapping, and interpretation [28]. Following initial interviews, a thematic framework was generated and discussed through an iterative process and informed by both the emerging findings and the realist review [22]. This framework was used to code and chart all interview findings into NVivo12/14 (Lumivero) and Excel (Microsoft). The populated framework was discussed between the research teams, until the final themes and subthemes were agreed upon.

**Figure 1:**
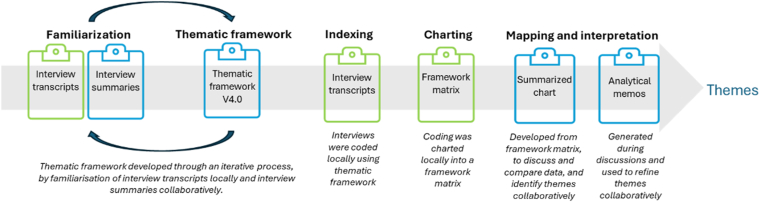
Process of framework analysis, following the 5 interconnected steps: (1) familiarization; (2) development of thematic framework; (3) indexing; (4) charting; and (5) mapping and interpretation of themes.

Throughout the analysis process, each research team analyzed their data in their respective languages throughout the data analysis process. A minimum of 2 researchers per team coded their respective datasets, alongside regular discussions within both local teams and between all research teams. Periodically, findings were summarized and translated into English to facilitate collaborative analysis processes, including regular discussions between all research teams. A detailed description of the analysis process, and how this was managed both at local level and collaboratively, is presented in Figure 1.

### 2.5 Ethical considerations

Ethical approval was obtained in each country: DK—The Danish Data Protection Agency through institutional registration (F2022-157); FR—French Research Ethics Committee (REC) Ile de France (ref: 23.00815.000198); SP–Ethical Committee for Clinical Research, Hospital Clinic Barcelona (ref: 2023-0336 ER-01); and the UK—London South East REC (IRAS: 323195; REC: 23/PR/0115).

Informed consent was obtained from participants. Data collection/storage was handled by each respective team. No personal, identifiable data were shared between teams.

### 2.6 Patient and public involvement

Two public contributors, in the UK and Denmark, contributed to the study design, including protocol and interview guide development. An additional contributor was recruited in the UK. Contributors in the UK reviewed and provided feedback on the thematic framework and the interpretation of themes. The lead public contributor is a co-author (K.S.) and a member of the patient and public involvement (PPI) task group for SERENITY [29]. PPI was planned and tracked via the Public Involvement in Research Impact Toolkit (PIRIT) [30]. Key PPI impacts are displayed in Supplementary File 1.

## 3 Results

**Figure 2:**
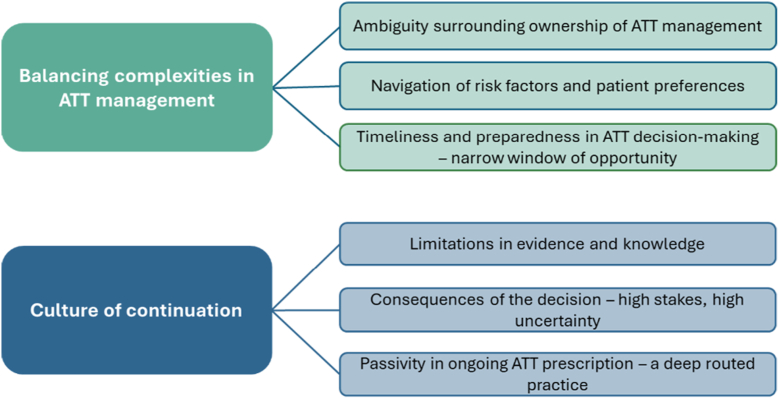
Themes and subthemes generated from framework analysis. ATT, antithrombotic therapy.

Eighty clinicians participated. Participants comprised palliative care doctors (*n* = 16; 20%); general practitioners (*n* = 9, 11%); oncologists, palliative care nurses, and vascular specialists—each represented 10% (*n* = 8); followed by cardiologists (*n* = 7, 9%), hematologists (*n* = 6, 8%), pneumologists (*n* = 6, 8%), geriatricians (*n* = 5, 6%), neurologists (*n* = 4, 5%), and internal medicine specialists (*n* = 3, 4%); 51% (*n* = 41) were men (Table). Interviews were conducted via videoconferencing (*n* = 65; 81%) or face-to-face (*n* = 15; 19%) and lasted 23 to 120 minutes. The following 2 themes were generated (Figure 2):

1. Balancing complexities in ATT management
2. Culture of continuation

**Table:** Participant demographics

Characteristic	All	Denmark	France	Spain	UK
Clinicians (*n*)	80	15	18	25	22
Male	41 (51)	5 (33)	12 (67)	12 (48)	12 (55)
Specialty					
Palliative care	16 (20)	4 (27)	3 (17)	6 (24)	3 (14)
General practitioner	9 (11)	2 (13)	1 (6)	3 (12)	3 (14)
Palliative care nurse	8 (10)	2 (13)	0 (0)	3 (12)	3 (14)
Oncology	8 (10)	2 (13)	2 (11)	2 (8)	2 (9)
Vascular medicine/surgeon	8 (10)	2 (13)	3 (17)	2 (8)	1 (5)
Cardiology	7 (9)	3 (20)	1 (6)	2 (8))	1 (5)
Hematology	6 (8)	0 (0)	2 (11)	1 (4)	3 (14)
Respiratory/pneumologist	6 (8)	0 (0)	2 (11)	2 (8)	2 (9)
Geriatrician	5 (6)	0 (0)	2 (11)	1 (4)	2 (9)
Neurology/stroke medicine	4 (5)	0 (0)	1 (6)	1 (4)	2 (9)
Internal medicine	3 (4)	0 (0)	1 (6)	2 (8)	0 (0)

France: home hospitalization clinician—categorized in palliative care (*n* = 1); internal medicine, in vascular medicine—categorized in vascular medicine (*n* = 1). Spain: general practitioner specializing in palliative care—categorized in palliative care (*n* = 3); geriatrician specializing in palliative care—categorized in palliative care (*n* = 2); internist specializing in palliative care (*n* = 1).

Selected quotes presented below illustrate the themes and subthemes generated, with representation across the breadth of clinical specialities, as well as country. Additional quotes are available in Supplementary File 2.

### 3.1 Balancing complexities in ATT management

#### 3.1.1 Ambiguity surrounding ownership of ATT management

Clinicians described ATT management as multidisciplinary, involving relevant specialists, such as cardiologists, hematologists, and oncologists; they believed ATT decisions were best made jointly, alongside the patient and their family.

“I think it has to be a joint decision. Firstly, we have a specialist who is dedicated to thrombosis … then there is the oncologist who is more involved with active treatment, to understand the prognosis of the [cancer] disease and the ongoing treatments, and of course, the patient’s wishes.” [SP08—oncologist]

Clinicians with established links to other relevant specialties described this as beneficial. However, some participants felt that specialized input was underutilized in practice, and they described working in isolation to other specialties.

“We’re in a silo medicine, where every specialist is in his corner.” [FR12—geriatrician]

This sense of fragmentation contributed to ambiguity around roles and responsibilities, with some questioning their expertise to lead ATT decisions, particularly for noncancer indications. Consequently, clinicians highlighted the challenges of deciding on ownership for ATT decisions.

“There’s ownership issues [ATT management], usually the deferral is either, if it wasn’t for a cancer specific related cause, there’s an antipathy I think in terms of us, initially anyway, to make that decision.” [UK05—oncologist]

Given the context of advanced cancer, many clinicians felt such responsibility would naturally fall under palliative and/or primary care. However, this was not always straightforward, and they acknowledged both specialist advice and close understanding of the patient and their current condition were essential to feeling confident in initiating and leading ATT decisions.

“If we know the patient well and have followed them throughout their cancer journey, then it should be us … but of course, if it’s a patient we haven’t been involved with at all during their cancer illness…. I think it can feel unsafe for both the patient and their family if a general practitioner suddenly comes in from the sidelines.” [DK07—general practitioner]

Clinicians described a reluctance to alter ATT decisions made earlier in the patients’ care, often placing considerable weight on the judgment of the original prescriber. This created a paradox, whereby clinicians deferred responsibility, assuming the original prescriber was better placed to make the decision. However, the original prescribers often did not feel deprescribing was their decision to make, resulting in inevitable decision inertia.

“Sometimes I have the feeling, this is subjective … that a lot of reliance is placed on the cardiologist's decision … and that sometimes bothers me a little.” [SP07—cardiologist]

Thus, ATT decisions were influenced by clinicians’ perception of their role and others’ roles, highlighting ongoing uncertainty about ownership and responsibility.

#### 3.1.2 Navigation of risk factors and patient preferences

Clinicians reported numerous complex factors influencing ATT decisions, many of which competing. This included bleeding and thrombotic risk, ATT indication, performance status, prognosis, and patient preferences.

“There are many factors in this when you think about the outcomes and what people want … some may want prolongation of life … others say symptom control … they don’t want another PE (pulmonary embolism) because that was an awful experience, or having a massive bleed is the worst nightmare … so, once you take all those different factors together, plus about 4 or 5 others that I haven’t thought of now, then you get a sort of quite complex mix, a complex algorithm….” [UK06—palliative care]

However, while clinicians concurred on the competing factors affecting ATT decision making, their perceptions of the impact of each factor varied. For example, some clinicians highlighted they rarely encountered certain events (eg, bleeding), and as such, these risks were less tangible to them.

“It’s not something that raises itself clinically for me … [but] one of the things that I noticed was feedback from other teams to say well, ‘you’re not seeing the bleeds on these drugs because we’ll sort it out’.” [UK18—cardiologist]

Overall, clinicians expressed more concern regarding thrombotic complications, over bleeding.

“If the patient undergoes a thrombosis and the thrombotic risk is very high, it’s going to kill him. If the patient has hemorrhage, he’s not going to die right away, I have time to react.” [FR18—hematologist]

Notably, in contrast to this, clinicians who had managed patients with a significant bleed while on ATT, described how these experiences shifted their prioritization in decision making toward bleeding risk over thrombosis.

“You may have managed very well along the way, and maybe at a specific moment there is a bleed and everything you have worked for a long time … ends up being the most horrifying moment.” [SP05—palliative care]

One of the key factors influencing ATT decisions, as described by the clinicians, was patient preferences. This became the deciding factor, when faced with the uncertainty surrounding other competing factors. However, some clinicians also expressed caution around the reliance on patient preferences, stressing the importance of not leaving the decision up to patients alone.

“I think there’s a real dilemma when professionals don’t dare to, or don’t want to, carry the responsibility of making a decision … I believe that the patient should always have the right to decide … [but] the professional has to take responsibility…. I completely agree that patients should be involved … [but] they shouldn’t have to make that decision themselves.” [DK15—palliative care nurse]

Hence, both competing, uncertain risk–benefit profiles and patient preferences were crucial elements of ATT decisions.

#### 3.1.3 Timeliness and preparedness in ATT decision making—a narrow window of opportunity

Some clinicians stressed the importance of early preparation for decisions relating to ATT among patients at the end of life, from changing the earlier messaging that ATT is lifelong, to the recommendation of timely advance care discussions and integration of palliative care to ensure patients are prepared and supported for such discussions. However, this was complicated by broader advance care conversations, particularly if the patient was unprepared or unwilling to discuss their prognosis.

“I think with anything like advance care planning, the earlier the subject is raised, you hope the smoother that [process] is, and you are not in a situation where the patient is acutely unwell and trying to have all of these conversations that are a reaction to an event. I think introducing the idea early would be helpful to prepare a patient … [but] I think it’s hard, if a patient hasn’t talked about prognosis and doesn’t want to.” [UK28—palliative care nurse]

As such, ATT decisions were just one of many medications or health concerns, which needed to be balanced and fit in with wider advanced disease-related conversations.

“In palliative care you have to proceed in stages, because there are other things to deal with than stopping antithrombotics … there are things that need to be managed beforehand.” [FR05—palliative care]

However, this left a narrow opportunity to engage in timely discussions surrounding ATT prescription with patients and their families. For example, some clinicians highlighted deprescribing ATT too early would risk thrombotic events, whereas too late could increase the risk of bleeding. Furthermore, decision making left until the last days of life removed a patients’ opportunity to be involved in the decision, since medications tend to be stopped at this stage by default.

“That’s part of the dilemma. But in a way, it also becomes easier—when the patient is clearly dying, then it’s no longer a difficult decision. You might not even need to reach the point where you overhaul the entire medication list, but just before that—that stage—things tend to become easier.” [DK15—palliative care nurse]

Prognosis was not only a prominent factor but also a challenge in ATT decisions. Clinicians reported ATT should be stopped once the harms outweighed the benefits, and when patients were very end of life, usually defined as the last days/week of life, and no earlier than 3 months. However, clinicians highlighted a great deal of uncertainty of defining both when the risk–benefit profile changes and when the patient was at end of life.

“It’s often unclear that they are in the last year of life, and there have been occasions when I’ve asked oncologists can you clarify what the prognosis here is, and they’re not usually very helpful or explicit in answering that question.” [UK18—cardiologist]

Instead, clinicians relied upon triggers such as bleeding, ability to swallow or administer medication, transition to palliative care, and de-escalation of active cancer treatment. However, the timing of the latter 2 were described to vary between patients.

“When we have stopped all active treatment, that is probably a good time for us to start rationalising … now that can happen at different time points for different patients.” [UK08—oncologist]

Therefore, ATT decisions were influenced by a delicate balance of end of life and multiple disease considerations, complexities of prognostication, and limited window of time for shared decisions to occur.

### 3.2 Culture of continuation

#### 3.2.1 Limitations in evidence and knowledge

Clinicians highlighted gaps in the evidence base guiding ATT decisions, describing existing evidence as irrelevant or poorly suited to the complexity and heterogeneity of cancer patients at the end of life. Limitations in risk prediction and quality of life tools were noted as particular concerns.

“The pendulum swings back and forth in the field of thrombosis and cancer: people are treated for the long term, and when there’s no more cancer, we stop [ATT]. But for people who are going to die of cancer, I don’t think it’s yet set in stone to systematically assess the benefit of treatment.” [FR16—pneumologist]

Some clinicians described the varying and sometimes limited understanding in their colleague’s approach to ATT management in the context of cancer, particularly when compared with management in noncancer contexts.

“There’s a lack of understanding, verging on ignorance on the part of healthcare professionals who are used to managing non cancer patients, or if they’re used to managing cancer patients in a very protocolized way.” [UK02—hematologist]

Clinicians also described the consequences of limited evidence on the ability to present information to patients in an understandable way, balanced with ensuring patients’ confidence in their clinical judgment.

“If we had more evidence based medicine, certainty about the risks, we would be more comfortable discussing it with the patient … sometimes you don’t have the answer. So it’s difficult, when you don’t have the answer to involve the patient.” [FR02—oncology]

Clinicians expressed the need for more support and guidance, particularly to be able to demonstrate that ATT decisions were reasonable and informed by the best possible evidence.

“We do just find it really difficult compared to other medications. Somebody will be on ten meds, they’re all stopped, but they’re left on their DOAC [ATT] 99% of the time. We’d be really pleased to have some sort of guidance on it.” [UK34—general practitioner]

Therefore, clinicians expressed the need for more evidence to support ATT decisions, to inform their recommendations.

#### 3.2.2 Consequences of ATT decisions—high stakes and high uncertainty

Clinicians described the varied and competing consequences of both continuing and deprescribing ATT. They described balancing these risks as challenging, with serious consequences on either side of the decision, particularly competing thrombosis or bleeding risk.

“We often have this kind of puzzle in oncology: if they bleed, we stop the anticoagulants or antiplatelets, but if we stop, they will be symptomatic on their pulmonary embolism.” [FR02—oncology]

Many clinicians described being more concerned about the decision to deprescribe than about continuing ATT.

“We are mainly focused on symptomatic control … ATT deprescription is more difficult for us. We worry about the risk if we withdraw it [ATT].” [SP20—geriatrician]

Clinicians also described feeling less supported or that it was less acceptable to risk the consequences related to deprescribing ATT. They expressed concern that if proactively stopping treatment was followed by a negative outcome, it would be judged more harshly than a negative outcome while receiving ATT.

“What you often have to navigate in real life is the understanding that if you make a proactive decision, and there is a negative outcome, even if that is an entirely predictable negative outcome and you believe that was a reasonable risk…. I think we’ve probably all had the experience where being entirely correct doesn’t shield you from the stress of attending to complaints and sometimes it could easily feel like the safest decision is no decision, [or] to make it someone else’s decision.” [UK12—geriatrician]

In relation to this, some clinicians highlighted their perception that patients and their families held strong preferences toward ATT. They described a hesitance to raise decisions about ATT if patients felt strongly about continuing and had been told it would be for life.

“If they have it in their mind that this is very important and they’re strongly convinced that it’s life-saving medication, then I don’t think I would correct them at this stage—not if I don’t believe it myself. I mean, assuming there aren’t any other red flags.” [DK01—cardiologist]

Hence, the consequences of initiating ATT review, particularly around deprescribing, were multifaceted.

#### 3.2.3 Passivity in ongoing ATT prescription—a deep-routed practice

For clinicians, the concept of stopping was counterintuitive, and they described being more comfortable continuing medications over deprescribing. ATT was distinguished as particularly challenging to consider deprescribing and was often the last medication stopped.

“They’re started on it, and then it just continues. And I don’t think anyone, myself included, really steps in. Because I wouldn’t even know if stopping it is the right thing to do, you know?” [DK09—vascular]

Clinicians described that unless there was a significant trigger, such as bleeding or difficulties with administration, ATT was continued with adjustments, or alternative options considered over that of stopping.

“There’s more of an effort to stop them in a way, because you have to sort of really talk to the patient about it … whereas it’s easier to maintain the status quo, of nice and safe, we’re on anticoagulation … let’s not rock the boat.” [UK06—palliative care]

As such, some clinicians highlighted a lack of appropriate triggers to initiate ATT review, outside of the above-described overt triggers.

“We’re without a trigger now, so I think unless there is some sort of trigger where people say, okay, the surprise question, or anticipatory medications, or, something to make the trigger of ‘should we continue’?” [UK03—palliative care]

In relation to this, clinicians described how ATT prescription tended to go unnoticed, with little thought spared for ATT decisions.

“I believe that the issue of anticoagulation is something that is undervalued, undertreated, and not given the importance it actually requires, and sometimes it even goes unnoticed.” [SP04—general practitioner]

Among ATT, antiplatelet agents were often overlooked, and at times, clinicians described uncertainty about the initial reason they were prescribed. In contrast, clinicians highlighted finding antiplatelet agents easier to discontinue, since their indication was usually preventative.

“I think aspirin is a very typical medication that often doesn’t get much attention … and it would be one of the first things you’d consider pausing if it’s only being used for prevention.” [DK03—oncologist]

Some clinicians were less reticent with the concept of ATT deprescription, although they acknowledged their colleagues were generally uncomfortable in this role.

“I’m at ease [with ATT deprescription]; it makes everyone else uncomfortable, that’s for sure, because it’s a dogma to leave it until the end.” [FR05—palliative care]

Therefore, many clinicians describe a passive, ingrained culture favoring ongoing prescribing of ATT, making it one of the last medications to be stopped.

## 4 Discussion

The challenges of managing ATT in the palliative care context is best illustrated by population data showing most cancer patients continue treatment until the end of life [5,7,8]. Previous research has attributed this practice to a discomfort among clinicians in the decision-making process, although the factors driving such perspectives have not been explored in detail [31]. Across the dataset and countries, clinicians described many complex influences on ATT prescribing in patients with advanced cancer near the end of life. While clinicians valued the involvement of other specialties, patients, and their relatives, they faced challenges in accessing the necessary support and expertise. This influenced their willingness to lead ATT decisions, with other barriers including a reluctance to override another prescriber’s decision, complex risk–benefit ratio, varying perceptions of ATT risk, and lack of familiarity with the patient. This was further complicated by 2 overarching contexts: ATT in end-of-life care and a general culture of drug continuation, leaving ATT as one of the last medications to be deprescribed, if ever. This culture is bolstered by lack of evidence, guidance, and tools to guide ATT decisions in this context and overriding concern of the consequences of deprescription.

Clinicians believed ATT decisions in this context should be multidisciplinary and include patients and their families. However, they reported variance in multidisciplinary input into ATT decisions. This supports previous research that multidisciplinary involvement, although not without challenges, is key to deprescribing but can be a barrier if health care is siloed, fragmented, and/or underdeveloped [19,20,32,33].

A key impediment to decision making was the ambiguity and lack of clarity surrounding health care professionals’ roles in the process. Palliative and primary care clinicians were considered by others and felt themselves to be best placed to lead, and initiate, ATT decisions at the end of life. However, their perceived role varied, with hesitancy to override the initiating specialist’s decision to commence ATT. This was further hindered by lack of familiarity with patients recently transitioned from oncology. These challenges are well described in the general context of deprescribing, reinforcing the importance of a trusting and continuous patient–clinician relationship to optimize deprescribing decisions [19,20,34].

Although clinicians’ perceptions of the risks of ATT in the context of advanced cancer and end-of-life care varied, more concern was attributed to the consequences of discontinuing over continuing. This aligns with other studies exploring deprescribing in the context of polypharmacy, where decisions are strongly influenced by clinicians’ individual risk conceptualization, which is often informed by previous experiences of deprescribing [19]. This aligns with our finding that clinicians who experienced a patient having a bleed on ATT, expressed strong convictions that complications of bleeding were worse than those associated with stopping ATT.

However, many clinicians interviewed reported having not seen any anticoagulation related bleeding events, which appeared to skew their stance on risk. In practice, bleeding was described as one of the most prevalent triggers to deprescribing ATT. Arguably, bleeding is not an ideal trigger, since the intention of deprescribing is to prevent bleeding complications, and it could be argued that it indicates a failure of timely ACP; however, although not ideal, it aligns with existing literature, suggesting that overt events are better triggers for deprescribing [19]. Ideally, suggested triggers such as transition to palliative care and ACP would serve as more appropriate and pre-emptive initiators of decision making, and these clinical milestones are less tangible and subject to considerable variability. Our findings reflect this variance in clinical pathways and transitions in care, complicating the ability to identify consistent and replicable triggers for reviewing ATT at end of life.

In the face of this variability, clinicians described patient preferences as a key consideration in ATT decision making. This aligns with existing research demonstrating patients’ involvement and preferences are crucial in deprescribing [33,35,36]. However, in practice, this may be challenging since patients have described a preference toward clinician-led decisions on ATT [23]. This potential disconnect may drive passivity in ATT decisions, as each await the other to initiate and trigger ATT review. The limited evidence base and paucity of guidance for clinicians, coupled with the variability of patients’ knowledge and understanding of ATT [23,[37], [38], [39], [40]], illustrate the challenges of offering an evidential basis for shared decision making.

In practice, making the time to discuss ATT decisions is considered challenging, particularly in the context of polypharmacy and advanced disease. Varied transitions in care, coupled with concerns over changing goals of care to a more holistic approach, often overshadowed focus on specific medication management. Failure to prioritize ATT review led to it being postponed, thereby narrowing the opportunity to dedicate sufficient time for meaningful discussion. These findings support the existing literature that describes the uncertainty surrounding optimal timing for deprescribing, which was identified as a key factor in the late initiation of palliative care and deprescribing in general [20,41]. To address this, clinicians advised informing patients early that ATT will need to be reviewed over time, potentially facilitating shared decision making later in the patients’ pathway and mitigating passivity associated with ATT prescription. This aligns with general deprescribing literature advocating for upstreaming these conversations and a planned, proactive approach to deprescribing [20,41].

The culture of continuation evident in clinical practice strongly influenced ATT decisions. Factors included clinicians feeling unsupported to deprescribe medications, resulting in ATT continuing by default. This reflects existing culture and practice [32] and supports calls to normalize deprescribing practices [20]. Our findings highlight that clinicians distinguished ATT from other medications as particularly difficult to discontinue, adding to the existing literature ATT represents a particularly complex class of medicines to deprescribe [33], within the context of an already challenging practice.

Recommendations based on findings from this study (Supplementary File 3) have contributed to the development of an SDST as part of the wider body of work within the SERENITY consortium [[5], [6], [7], [8],[22], [23], [24]], which will be evaluated in a cluster randomized controlled trial [21]. Additionally, these findings provide important insights into the challenges and considerations related to ATT management beyond informing the SDST. Our findings captured perspectives across a wide range of specialties, highlighting the complex interplay of interdisciplinary and individual clinicians’ roles and responsibilities, patient preferences and risk perceptions, timing of decisions within the context of advanced cancer at end of life, and general and specific culture of continuation pervading on ATT decisions. Future exploration of the specific nuances both between and within specialties and countries would further elucidate the impact of the varying roles and responsibilities different clinicians have on ATT management and decisions among this patient population.

### 4.1 Strengths and limitations

A key strength of this study is the large and varied sample of experienced clinicians, alongside the iterative sampling approach, to ensure data were captured across a breadth of European countries, clinical specialties, health care settings, and cultural contexts. From this, we have achieved a broad understanding of ATT management, which may be transferable across a wider range of settings and practice. The application of our findings in wider practice should still be considered with caution since these data were collected in Northern/Western and Southern European countries, and our findings may be of less relevance to different health care systems. The research team included a wide range of multidisciplinary expertise, including clinical and qualitative experience, and this variance likely influenced data collection. However, a comprehensive protocol and interview guide were standardized and adhered to, alongside regular meetings to mitigate this variance. The use of Framework Analysis proved to be suitable and flexible for analysis of such a large dataset across multiple locations.

## 5 Conclusion

The management of ATT in cancer patients approaching end of life is multifaceted and influenced by several, often competing factors. These complexities need to be understood and addressed to support decision making related to ATT at the end of life. In practice, there needs to be an approach that not only improves or facilitates clinicians’ confidence in approaching ATT decisions but is also a way to present it so that it is understandable and acceptable to patients. There are many possible approaches to addressing these challenges, of which an SDST is one. Irrespective of which approach the profession eventually takes, it is essential that it is subject to robust prospective evaluation. Doing nothing and maintaining the status quo is no longer considered an option by the authors.
