# Supplementary material for: Clinician perspectives on antithrombotic therapy management in advanced cancer: a multinational qualitative study
Source: Res Pract Thromb Haemost. 2026 Mar 25;10(3):103427. doi: 10.1016/j.rpth.2026.103427 (PMC13092592; doi:10.1016/j.rpth.2026.103427)
Supplement: Supplementary File 1 [file mmc1.docx]

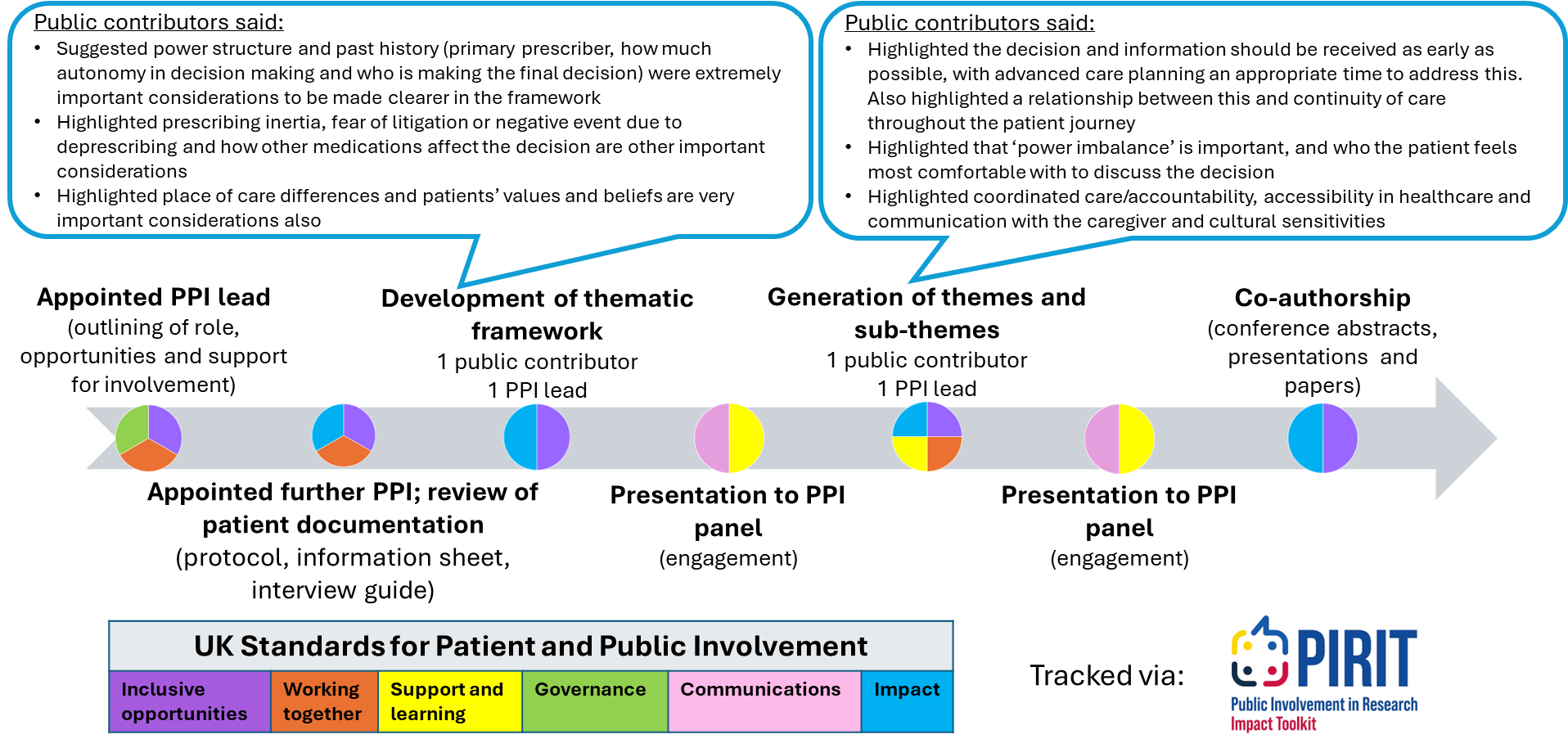
Supplementary File 1: Patient and public involvement, engagement and impact

***Fig. legend:*** *overview of patient and public involvement, engagement and impact throughout the study. Within the speech bubbles are evidence of some key feedback from public contributors during data analysis, and following a timeline of activities, which have been mapped to the UK standards for Patient and Public Involvement.*
