# Supplementary material for: Clinician perspectives on antithrombotic therapy management in advanced cancer: a multinational qualitative study
Source: Res Pract Thromb Haemost. 2026 Mar 25;10(3):103427. doi: 10.1016/j.rpth.2026.103427 (PMC13092592; doi:10.1016/j.rpth.2026.103427)
Supplement: Supplementary File 2 [file mmc2.docx]

**Supplementary file 2 Table of additional quotes V1.0 28.10.2025**

**quotes in bold represent the quotes present in the articles’ main body of text*

**Theme 1: Balancing complexities in ATT management**

**Sub-theme 1.1:**

| Sub-themes | Essence point | Country | Quotes |
| --- | --- | --- | --- |
| Ambiguity surrounding ownership of ATT management | ATT management requires multidisciplinary input (inc. clinicians and patient) | DK | "If the general practitioner has the authority and feels that he can make the decision through a shared decision-making process with the patient—again, a common decision-making basis—then I think he can go ahead and do it. And it should be such that he can contact us at the anticoagulant clinic for advice and guidance—'What do you think?'—and then I think I would take the same approach here. I believe I would be quite open to saying they should stop.” [DKC10 - cardiology] |
|  |  | FR | “It's a collegial decision, it can't be taken alone… I have a principle in palliative care, if you decide to stop, you don't take the decision alone, you discuss it with the other doctors who are following him and I also discuss it with the patient.” [FRC3 – general practitioner] |
|  |  | SP | **“I think it has to be a joint decision. Firstly, we have a specialist who is dedicated to thrombosis… then there is the oncologist who is more involved with active treatment, to understand the prognosis of the disease and the ongoing treatments, and of course, the patient's wishes.” [SPC8 - oncologist]** |
|  |  | UK | “It’s that risk-balance and we do see more of those conversations, definitely over the last few months, there’s a few difficult ones that kind of come to mind, and obviously it’s very multidisciplinary at that point… it can be seeing haematologists out of hours, obviously getting CAT [cancer associated thrombosis clinic] input as well. It can be quite complex, but it seems very multidisciplinary in the conversations.” [UK27 - palliative care nurse] |
|  | Benefits of multidisciplinary structure/ ATT decisions are multidisciplinary | DK | "We have a very close collaboration with the oncologists, and we really have a lot of contact with them—both us and the nurses. So I think it becomes... I mean, I think it’s different in some way. I really think it is.” [DKC2 - cardiologist] |
|  |  | FR | “It’s always quite complicated because, as I said, we always have the thromboembolic risk behind our heads. but then it’s a collegial discussion, and indeed, when the end of life approaches, we try to focus on quality of life, so it’s not a discussion that we will have alone, we do it in collegiality with people who take care of the patient. And then we take the advice of the palliative team, and our opinion too, on I mean the advice of colleagues, we really discuss with, we get closer to vascular.” [FRC14 - geriatrician] |
|  |  | SP | “A multidisciplinary team is always the best for the patient, where everyone can contribute their bit in the field in which they are most specialized." [SPC1 - internal medicine] |
|  |  | UK | “I am fortunate I’ve got colleagues whom I know so well, we get along very well with palliative and haematologists both actually. And in our health board, they do respond within a day, within hours I should say. I think having that on-hand linkage helps.” [UK13 - geriatrician] |
|  | Multidisciplinary input is not consistent in practice; there is isolation between specialties they would like to involve in ATT management | DK | "I mean, it would be really good to have collaboration with the professions involved. I imagine that includes the thrombosis centre, cardiologists, and neurologists. But we’re not prepared for that yet. Still, if we collaborate, it might work. It could also fall under the cardiology department, where they can provide recommendations from their side." DKC3 – oncologist] |
|  |  | FR | **“We’re in a silo medicine, where every specialist is in his corner.” [FRC12 - geriatrician]** |
|  |  | SP | "Well, and that sometimes you feel quite alone in making these decisions… less with the hospital, but yes with primary care, we usually talk to the responsible doctor, and we do reach a consensus. With oncology and other hospital services, sometimes there is less access, right? Due to overload or because we don't have a direct communication channel, but with primary care, yes, I wouldn't say always, but maybe half the time we do discuss it, right? But in the end, the family doctor says, 'Whatever you think, whatever you consider...'" [SPC12 - palliative care] |
|  |  | UK | “I think there is a role, yeah I think there is a role again erm, I suspect also erm, I mean a person like you, um, you are a perfect decision-maker from the haematology point of view, as well as palliative care. But most people are not. So, I think we need to use the knowledge and expertise of our haematologists perhaps more when we make these decisions, and we don’t at present.” [UK14 - respiratory] |
|  | Ultimately, while multidisciplinary input is needed, there can only be one responsible clinician to lead on ATT decision; however, expertise is important | DK | “It’s still a collaboration with cardiologists. They work a lot with rhythms and risk assessments, and they have different types of scoring systems. But they don’t really take the oncological part into account — apart from just noting that the patient has cancer. So there’s a lack of nuance in how the patient’s situation is understood.” [DKC3 – cardiologist] |
|  |  | FR | “Honestly, cardiologists don’t care. I think it’s the thrombologist we’ll call or the general practitioner. But I think we’ll just ask the cardiologist for advice: knowing the cardiologist, he’ll say we touch nothing we leave the anticoagulant. The problem with my cardiologist friends is that they always tend to say, we leave the anticoagulant until death. I think it is unfortunately not a single doctor, I think it is the doctor of his palliative care associated with the cardiologist and I in my opinion it is a new profession that was invented: the thrombologist, the one who masters the anticoagulants, here I would do it a little collegial way.” [FRC15 - vascular] |
|  |  | SP | “I believe that the most appropriate approach would be to have a multidisciplinary team. The patient could be followed throughout the final stage of their life by the palliative care specialist and at the health center with all associated resources, including the liaison nurse and the family doctor. However, decisions regarding anticoagulation or antiplatelet therapy could be addressed through a specific consultation for evaluation, a dedicated consultation for this issue.” [SPC1 - internal medicine] |
|  |  | UK | “It’s a multi-disciplinary sort of thing but generally, ultimately, it’s the person co-ordinating all of that as an individual overseeing their [the patients’] care [who’s responsible].” [UK29 - haematologist] |
|  | There is lack of clarity and ambiguity surrounding roles and responsibilities for ATT management | DK | "Well, you could say that, on paper, it’s probably the general practitioner who officially has the responsibility. But in reality, it’s probably mostly us who end up taking it on. Technically, we’re just consultants, although we’re very closely involved with the patients. We usually say that we’re only involved in the palliative treatment. But when it comes to the final stage, we often have such close contact with them that we more or less take over the treatment responsibility—informally, really.” [DKC11 – palliative care] |
|  |  | FR | “After the diagnosis of cancer, patients are phagocytized in fact, after which they are no longer seen, or may be at the end of their life because they no longer have treatment, or have stopped treatment, if they can no longer move about and wish to remain at home - for example, a patient with lung cancer, who has a very steady rhythm of chemotherapy. Since then, we've stopped seeing her, we've just stopped seeing them. They certainly have cardio monitoring, for example, with the chemo, but we don't see them anymore.” [FRC3 - GP] |
|  |  | SP | ''It's just that normally I handle them [CANCER PATIENTS ON ATT], but the oncologist always manages them too for the oncological disease part. I handle it for the thrombus part, to decide if... I don't know... if a patient has been on heparin for a while and we see that they are not undergoing active [ONCOLOGICAL] treatment now, and we think we can switch to oral anticoagulants, or if we can take them off... which is rare, so we decide together with oncology, and they consult with me, and I keep track of them.'' [SPC17-Internal medicine] |
|  |  | UK | **“There's ownership issues [in ATT management], it's quite interesting, usually the deferral is either, if it wasn't for a cancer specific related cause, there's an antipathy I think in terms of us, initially anyway, to make that decision.” [UK05 - oncologist]** |
|  | Clinical practices vary – responsible clinician can vary in the patient pathway within and between specialties, facilities and countries | DK |  |
|  |  | FR | “Ideally do this in pairs with either your GP or the one who originally prescribed the anticoagulant. if it’s the cardiologist, if it’s the GP. so it’s ideally to manage with him. but unfortunately in everyday life experience, patients stop seeing their GP at the time they have cancer, which is not good.” [FRC2 - oncology] |
|  |  | SP | “Very few patients are followed-up up to the end. These are mostly taken by oncology or by the palliative care team. And then we also have the home palliative care team.” [SPC17 - internist] |
|  |  | UK | “I am involved pretty much with my patients until the end of their life, because of the nature, where I work, I work in a standalone cancer centre, so patients, particularly in my patients with bladder and prostate cancer if they are bleeding they don’t come to our hospital, that’s not why they are admitted.” [UK08 - oncologist] |
|  | Palliative care and primary care are best placed to lead on ATT decisions in the context of advanced disease and end of life; but with the caveat of having support and expertise | DK | "I actually think — and this is something I feel quite strongly about — that we sometimes underestimate how skilled general practitioners are. And I honestly believe that palliative care teams could handle it too.” [DKC10 –cardiologist] |
|  |  | FR | “Under APA, when you have a palliative care vision, you have to leave them alone for the last month. if they have a month to live, I stop. then I don't bother. Once they're in palliative care, nobody takes care of them, and that's that. Sometimes it's the general practitioner. Who should do it? It's the people in charge of the patient, because palliative care is, by definition, all-encompassing, and that's where it should come in.” [FRC5 – palliative care] |
|  |  | SP | "The one who has to make the decision is the one who knows the patient best. I think this is the... obviously, a consensus can be reached with the palliativist or the haematologist or whoever is appropriate, with the expert, right? But I believe that the one who has to make the decision, or the doctor who is responsible for this patient, is their primary doctor, the one who has always taken care of them. Although then the oncologist... may have a specialist who deals with the thrombosis, who can also provide their opinion or multidisciplinary view, but I think the one who has to make the decision or who knows what the patient's life expectancy is, in quotes, or how the patient is clinically, is the doctor who regularly sees the patient." [SPC11 - haematologist] |
|  |  | UK | “I would say the general practitioners would be, on that list… but I would imagine you know generally one of them would perhaps have an interest in palliative care. So a GP with an interest in palliative care, a cardiologist who deals with anticoagulation on a regular basis. Those sort of people.” [UK23 - stroke consultant] |
|  | Equally important is familiarity with the patient; recent transitions to different clinicians can complicate clinicians’ willingness to take on the role in ATT decisions | DK | **"I think that if we know the patient well and have followed them throughout their cancer journey, then it should be us… but of course, if it’s a patient we haven’t been involved with at all during their cancer illness — and there are some whose cancer progresses so rapidly that we barely even get a chance to connect with them before they’re no longer in active treatment — then I think it can feel unsafe for both the patient and their family if, say, a general practitioner suddenly comes in from the sidelines and says, ‘Now you shouldn’t take this medication anymore.’**  **I think it should be the doctor who has been following the patient’s course, really. And I believe we can take that responsibility — and I think the palliative care team can take that responsibility too. The most important thing is that the patient feels safe with the person making the decision." [ID7 – general practitioner]** |
|  |  | FR | “I'm not an oncologist, so I don't deal with the cancer part at all. on the ATT side, I'm really the one who's going to make the decision. but we're going to talk about it. it's important to be able to talk about it, precisely because they often know the patient better than we do. overall, they see the patient more often than we do, and they know the people around him better, etc., which is useful for making decisions.” [FRC17 - internal med, vascular] |
|  |  | SP | “I believe the one who has to make the decision, or the doctor who is responsible for this patient, is their primary doctor, the one who has always taken care of them. Although then the oncologist... may have a specialist who deals with the thrombosis, who can also provide their opinion or multidisciplinary view, but I think the one who has to make the decision or who knows what the patient's life expectancy is, in quotes, or how the patient is clinically, is the doctor who regularly sees the patient." [SPC11 - haematologist] |
|  |  | UK | “I think whoever is familiar with the clinical circumstance of the moment to make it. Um, so for example, um, the CAT clinic is, they may send a letter saying this person only needs anticoagulation for six months say, um, because they’ve had a, you know, a femoral DVT or something, and then they can stop their anticoagulation. And you look at the leg, you look at the person, you look at the fact that they’re still having treatment, or they’re not having treatment but their disease is worse and they’ve got more pelvic obstruction and you think, I think it might be sensible to carry it on. Um, so it’s not always a discontinue, sometimes it’s actually, actually going back to them, I think it might be good, appreciate I’m seeing this person and you’re not, therefore you wouldn’t know these factors but shall we, shall we carry on? Or I chat it through to the GP and the GP says, yeah, happy to prescribe, don’t worry about it, we’ll keep a little beady eye on this between us.” [UK24 - palliative care] |
|  | Specialist access could be inconsistent and clinicians may not feel they have the familiarity and have all the information to lead on ATT decisions | DK |  |
|  |  | FR | “Under antiplatelet agents, when you have a palliative care vision, you have to leave them alone for the last month. if they have a month to live, I stop. then I don't bother. Once they're in palliative care, nobody takes care of them, and that's that. Sometimes it's the general practitioner. Who should do it? It's the people in charge of the patient, because palliative care is, by definition, all-encompassing, and that's where it should come in. [FRC5 – palliative care] |
|  |  | SP | "Well yes, especially at times... it causes me conflict because usually the responsible doctor prior to our arrival doesn't tend to discuss these topics with the family, even oncology, and they usually leave us with the hot potato, so to speak. 'Let the palliative care doctor decide,' right? [...] I've known the patient for 15 days, and you've been seeing him for a year and a half.” [SPC12 - palliative care] |
|  |  | UK | **“We look more to the oncology services, and hoping that they’re making a lot of the decisions… I don’t feel quite so much ‘in the loop’. I feel as if everything has become so sub-specialised that… they [the patient] have got the links sort of centrally. And a lot of the decisions are being made for us, which is great. But then we still end up being there maybe towards the end and still having to consider it [ATT].” [UK35 - GP]** |
|  | Reluctance to override the decision made by the primary prescriber of ATT / oncologist and/or ATT specialists’ opinion holds more weight | DK | "The challenge is often that the cardiologists — who primarily manage these medications, along with the neurologists to some extent — have very clear scoring tools and strong opinions that patients should be on antithrombotic treatment almost at any cost. I’m exaggerating a bit here, of course." [DKC13 – palliative care] |
|  |  | FR | “The oncologist has a weight in this kind of discussion, he is more listened to by the patient than anyone else. So my answer is the oncologist because he's the one who has a privileged listening with the patient, not because he is the best.” [FRC10 - home hospitalisation clinician] |
|  |  | SP | **“Sometimes I do feel... sometimes I have the feeling, this is subjective eh..., that a lot of reliance is placed on the cardiologist's decision, that it's like 'what the cardiologist says is sacred' and that has to done yes or yes, why? Because the cardiologist said so. And that sometimes bothers me a little.” [SPC7 - cardiologist]** |
|  |  | UK | “Because I am not the primary prescriber of it, I feel less comfortable, doing that. But I anticipate it’s hard for GPs to do it as well because maybe they don’t have as much insight to maybe how end of life they are, so they are probably waiting for a cue from me to say actually this is where we are, let’s rationalise their medications. I don’t necessarily do that, I have more insight where we are maybe in their cancer, in their life span and coming to end of life.” [UK08 - oncologist] |

**Sub-theme 1.2**

| **Sub-theme** | **Essence point** | **Country** | **Quote** |
| --- | --- | --- | --- |
| Navigation of risk factors and patient preferences | There are many, different and complex factors to take into consideration in ATT management *(bleeding, thrombosis, ATT indication, patient preference, patient performance)* | DK | Is the patient receiving anticoagulation for an acute thrombosis, or is it preventive treatment? There's a huge difference. And where I’ve often run into trouble is actually with patients who have atrial fibrillation and cancer at the same time. For example, when they can no longer swallow tablets — in those cases, I’ve sometimes, and I’ll openly admit this, probably been too aggressive with continuing preventive treatment.  It’s difficult to figure out, because the anticoagulation is only being given to prevent a thrombosis-related death, while the patient is in a terminal phase of their cancer and experiencing bleeding complications. And then you're faced with weighing what's most important… if there’s an active, major thrombosis, I tend to be more persistent about keeping them on anticoagulation. I think that’s the case for many of us. The problem is, it was once an active decision — but as the patient’s disease progresses, that decision doesn’t get reevaluated. It just continues passively… remaining life expectancy is also crucial — are we talking about tomorrow or several months? And then there’s the indication for the medication, and their quality of life." [DKC9 – vascular surgeon] |
|  |  | FR | “It’s really a discussion with the patient and there is no rule. I think that the recent or not indication of the AC treatment I think of the curative doses is a patient who has had a thromboembolic venous event, if it was last week, it’s not the same as if it was 6 months ago. to stop the AC treatment with curative dose in someone who made a PE last week, we are a little more bothered than if it is a person who did the same thing 6 months ago where we are less bothered to stop the treatment or decrease the dose. and then also, it depends a little bit on the PE because it is a very small distal PE, sub-segmentary or what it was a massive PE with the patient who spent 2 days in intensive care.” [FRC6 - pneumologist] |
|  |  | SP | "It also depends on the patient, but it depends on... sometimes it depends on the lesions. If I see a lesion that has a high risk of bleeding… even though they were not bleeding at the moment, I know that if they do bleed, they have a high risk of bleeding a lot. So sometimes, in such cases, I suspend the anticoagulation, as long as... for me, it depends a lot on the reason they are anticoagulated, but sometimes I don't suspend it. Instead, I lower the dose and leave them on a prophylactic dose of anticoagulant, as a middle ground. [...] If the patient is in good general condition, even if they are not undergoing active treatment, maybe they stopped treatment because it is an advanced stage and there is no other line of treatment. But if the patient is in good general condition, with a good performance status, I continue the anticoagulation. I wouldn't want to let them have an event because there is a high risk of an event if they are not anticoagulated between the cancer and the valve. So, in this case, if the patient is doing well, I continue it. If I see that they are doing very poorly, with a low performance status, with a Karnofsky score below 40, and entering the end of life, I consider stopping it. It depends on this, on the diagnosis, the quality of life, and the performance status they have at that moment." [SPC6 – palliative care] |
|  |  | UK | **“I think there are many factors in this when you think about the outcomes and what people want… some may want prolongation of life at all cost... others say symptom control… you have to base it on that, so they don’t want another PE because that was an awful experience, or having a massive bleed is the worst nightmare… so you balance it against those things. Plus, then you have to bring in the fact of, whether they’re having the medications as injections or by mouth… so, once you take all those different factors together, plus about 4 or 5 others that I haven’t thought of now, then you get a sort of quite complex mix, a complex algorithm…” [UK06 - palliative care]** |
|  | However, perception of risk factors could vary; there was also a sense of not being exposed to the ‘full picture’ in relation to risks in ATT management | DK |  |
|  |  | FR | “There are both complications: either a thrombotic event (a pulmonary embolism) or a bleeding complication. But the bleeding complications, we don’t see them much at home. they end up in gastroenterology. so we see because they have an acute coronary event or pulmonary embolism.” [FRC9 - cardiologist] |
|  |  | SP | “I practically do not see haemorrhagic complications. I mean, I recall of it in an anecdotal way as they usually enter the emergency room, we rarely see them unless we are consulted.” [SPC19 - vascular] |
|  |  | UK | **“It’s not something that raises itself clinically for me but again it’s one of those interesting things… one of the things that I noticed was feedback from other teams to say well, ‘you’re not seeing the bleeds on these drugs because we’ll sort it out’.” [UK18 - cardiologist]** |
|  | There is more concern relating to thrombosis risk over bleeding risk | DK | "Well, the risk is obviously bleeding, right? I mean, their vessels are—or tend to be—quite vascularized with a lot of blood vessels. So bleeding is a concern. It also depends a bit on where the cancer is located, and that could also lead us to consider giving aspirin instead of clopidogrel, because we do have patients... We already assess in advance whether the patient is high risk for bleeding. That includes whether they've had a previous bleed or if they've had a stroke or TIA, right? I don't think we do it systematically, but off the top of my head, I would consider a cancer patient to also be high risk for bleeding, and therefore one might consider giving aspirin instead of clopidogrel. But that wouldn’t stop me from prescribing an anticoagulant. No." [ID9 – vascular surgeon] |
|  |  | FR | **“If the patient undergoes a thrombosis and the thrombotic risk is very high, it's going to kill him. if the patient has haemorrhage, he's not going to die right away, I have time to react to treat him for haemorrhage, I have time. thrombosis, I don't have time, it's a much faster killer. So if it's a high bleeding risk, but I have the means to compensate for bleeding losses, I accept that, I take a risk, and I continue.” [FRC18 - haematologist]** |
|  |  | SP | ''...if a patient has been on heparin for a while and we see that they...can switch to oral anticoagulants, or if we can take them off... which is rare...'' [SPC17-Internal medicine] |
|  |  | UK | “That's the issue, and it may be sort of, you know, in it's not evidence based that sort of anecdotal, um, no, I don't see those as major issues in, in the patients that I have in terms of bleeding, now, you know, the group of patients I have, do have bleeding, so rectal cancer obviously being a common one, bowel cancer being common. These are presenting symptoms of patients with this disease, and that's not obviously common in breast cancer and other things, so I happen to work in a disease where this is more an issue. Having said that, if I've got somebody who’s got active bleeding and there isn't a strong reason for them to be on their, aspirin or non-steroidal, I will actually say, maybe stop that for a bit, but it's not, it's not a major discussion point, and again, that may be just a small number of patients who are on it, so I'm not noticing it as much.” [UK05 - oncologist] |
|  | Some clinicians had more concern/ awareness about bleeding, based on experience of a patient having a bleed | DK | “For us professionals, is that question of: “I wonder how long this patient will live?” I mean, what’s the greatest risk here? … And we might sometimes feel—well, the dilemma is that you don’t necessarily die from a blood clot, because then one might be tempted to think that dying from a clot is better than from a hemorrhage, simply because bleeding can be so violent. Or at least it can be. So that’s something that might be at play. I also experience—though I’m not quite sure if this is what you’re asking—but I also experience that for some home care nurses, this [bleeding] is more of an issue. Because they’re the ones who get the calls like, “Is now the time to have some syringes at home?” And what they mean is: Should we have something calming on hand, so we can help the patient sleep if they start bleeding… sometimes the home care nurses actually get scared and call us, saying “Is it now? What do you think?” But we also go out to patients. I remember a situation with one of our very experienced doctors—we were talking before heading out, wondering, “Is this the time to talk about the dark towels, so you don’t see the blood?” [I’ve told a caregiver that] if suddenly [the patient] starts bleeding a lot, she will lose consciousness very quickly. But it would be really, really good if you could sit behind her and hold her, so she feels she’s in good hands.” I didn’t tell him it was so he wouldn’t see the blood. And he actually did that. He ended up sitting behind his mother as she died from a massive hemorrhage. That’s also a story I’ll never forget, because I thought—wow. I also did a follow-up visit afterward. And he—well, all things considered—seemed okay. But it must have been incredibly intense. [DKC15 – palliative care nurse] |
|  |  | FR |  |
|  |  | SP | “You may have managed very well all along the way, and maybe at a specific moment there is a bleed and everything you have worked for a long time, the experience changes [due to an acute event] and ends up being the most horrifying moment.” [SPC5 - palliative care] |
|  |  | UK | “When we usually recognise they’re starting to actively die. And there’s no prol-, there’s no reason to prolong anything, there’s no reason to give them more of a burden of bleeding.” [UKC30 – palliative care nurse] |
|  | Patient preferences became a significant factor in ATT decisions at end of life – in the face of uncertainty surrounding the other factors | DK | “If they’ve been told elsewhere, or if they have it in their mind that this is very, very important and they’re strongly convinced that it’s life-saving medication, then I don’t think I would correct them at this stage — not if I don’t believe it myself. I mean, assuming there aren’t any other red flags.” [DKC1 – cardiologist] |
|  |  | FR | “It depends on the diseases, it depends on the patient, his context and his autonomy. So there are other criteria that need to be addressed in this decision. it is the polypathology, it is the entourage, it is the comfort of life, it is autonomy, it is the cognitive status.” [FRC12 - geriatrician] |
|  |  | SP | **"Their wish (patients'), for me, is very important because their wish concerns their body and it is their decision about a lot of things they want or don't want to continue doing. For example, if a patient openly tells you, 'I'm not afraid of dying by drowning, but I'm terrified of bleeding and dying from blood loss,' I believe that in such a case, the patient's decision takes precedence." [SPC6 – palliative care]** |
|  |  | UK | “So I think if someone’s entering their final days, and we’ve started the care decisions, or the care’s being guided by the care decisions, um, we will think about stopping that. Unless the patient’s got a strong wish to continue, um, and also depends on what the kind of sites are like as well, so um, those are the things that are possibly being reviewed as well.” [UK27 - palliative care nurse] |
|  | However, there is a balance of not relying on the patient to make that decision alone/ leaving the decision solely up to their preferences and overburdening them | DK | “I think there’s a real dilemma when professionals don’t dare to, or don’t want to, carry the responsibility of making a decision on behalf of the patient… I believe that the patient should always have the right to decide… But still… the professional has to take responsibility… I completely agree that patients should be involved… [but] they shouldn’t have to make that decision themselves. Because when you phrase it as a question, it suddenly becomes the patient’s burden." [DKC15 – palliative care nurse] |
|  |  | FR |  |
|  |  | SP | “I don't like paternalistic medicine. What I like is for the patient to know that the results are a matter of probability and that, therefore, there are alternative paths. Rarely is there only one valid option. So the patient needs to know... needs to be able to... also have some margin of decision because with cancer, it is very common to feel, quite rightly, that the cancer controls you, that you don't manage the progression. So, I usually ask for opinions. I usually offer one option, another, and I usually ask what they think. [...] I think almost all the decision is mine, but there is a margin where it is very important that the patient wants you to treat them.” [SPC2 – oncologist] |
|  |  | UK | “I always make it clear to them what my opinion is, so they don’t feel like I’m expecting them to make the decision. I would always give them, this is my opinion, but what you think is very important, so that if they essentially don’t want to feel like the burden of responsibility is on them… I don’t want them to feel like I’m pressuring them to make the decision. That’s all. I have essentially have no agenda of my own. I regard myself as kind of a butler almost, a sort of medical butler. Erm, the issue is, if all I do is lay out the facts and then say, what do you think, it feels like I’m asking them make the decision, and I don’t think that that’s fair, erm, but it’s not about me obviously trying, trying to promote my view, it’s to try and, erm, it’s try and not make people, people feel pressurised to make decisions about difficult things, sometimes.” [UKC12 – geriatrician] |

**Sub-theme 1.3:**

| **Sub-theme** | **Essence point** | **Country** | **Quote** |
| --- | --- | --- | --- |
| **Timeliness and preparedness for ATT decisions in the advanced disease context – narrow window of opportunity** | Being prepared / preparing patients earlier (and changing the messaging around ‘continuation is for life’, and importance of highlighting it as an ongoing process) | DK | "I think it’s also partly about whether they’ve been prepared a bit by the departments they’re coming from — so that they already have some idea of their disease status and prognosis, and we can kind of pick up that thread.  We probably can’t always look at things in isolation within our own department. It’s also about what kind of knowledge they bring with them when they come to us." [DKC8 - oncologist] |
|  |  | FR | anticipation, starting to explain to patients where we're going and where we're going from here, is an important factor, and for these decisions it's also important to hear from loved ones or to talk to us, to help the patient make the decision [FRC13 - oncologist] |
|  |  | SP | ''There are times when they drag that along, there is no... a consensus on the treatment to follow. Reconciliation, right? We would say, there is no reconciliation of that treatment. So, it seems like they accumulate, accumulate, and it comes to us and we have to start removing.'' [SPC23 - palliative care nurse] |
|  |  | UK | it’s a good time to have those thoughts isn’t it, because. You, if you leave it any later, you might erm, you might be doing them a disservice if you don’t consider. All the factors, yeah. [UK29 - haematologist] |
|  | The importance of preparing and being prepared for ATT decisions by the end of life stage | DK | "I think it’s also partly about whether they’ve been prepared a bit by the departments they’re coming from — so that they already have some idea of their disease status and prognosis, and we can kind of pick up that thread.  We probably can’t always look at things in isolation within our own department. It’s also about what kind of knowledge they bring with them when they come to us." [DKC8 - oncologist] |
|  |  | FR | “Anticipation, starting to explain to patients where we're going and where we're going from here, is an important factor, and for these decisions it's also important to hear from loved ones or to talk to us, to help the patient make the decision.” [FRC13 - oncologist] |
|  |  | SP | ''it is the... the talk, the dialogue, the conversation, the interview with the patient and their family, a bit looking at their preferences, their history, their biology, their beliefs, their fears, their weaknesses a bit as well, and seeing and explaining... it is a progressive process, little by little, where you start talking about the illness, the course of the illness, and addressing different complications that may arise. Above all, having trust and dialogue with the patient. Also with the family, but especially with the patient, according to their... a bit getting to know their biography and their fears and their... and their expectations as well.''[SPC11 - palliative care physician] |
|  |  | UK | “I think with anything like advanced care planning, the earlier the subject is raised, you hope the smoother that [process] is, and you are not in a situation where the patient is acutely unwell and trying to have all of these conversations that are a reaction to an event. I think introducing the idea early would be helpful to prepare a patient… [but] I think it’s hard, if a patient hasn’t talked about prognosis and doesn’t want to.” [UK28 - palliative care nurse] |
|  | Being prepared and initiating discussions about ATT specifically must be balanced with advanced disease and wider conversations about a patients health | DK | "In an ideal world, this would definitely be part of the conversations we have at the very first consultation with a patient — from my perspective. But from the patient’s perspective, I’m not so sure it always fits in, because there’s an overwhelming amount going on during that hour and a half we spend together: we talk about finances, symptoms, and questions like 'What will happen when you die?', 'Will you be going to hospice?', and then on top of that, we also need to remember to switch the paracetamol to as-needed dosing, and so on.” [ID13 – palliative care] |
|  |  | FR | Compared to our European colleagues there is a difference: for most oncologists, the end of life is the last 15 days or the previous month. and I think that in France a mistake commonly made because we have not prepared the people or their relatives I completely adhere to the fact that when we begin to estimate that in a year, "it will be goodbye Berthe", we must still begin to think about after. after telling the patients, and sometimes I warn them earlier when they are metastatic, about the fact that there will be no cure, it is necessary to prepare I will prepare very upstream [FRC13 - oncologist] |
|  |  | SP | Also, the palliative care team should be very integrated from the beginning, not just called in when the patient is no longer receiving chemotherapy or treatment, which might give the patient the feeling that they are being abandoned, in quotes, and that all we are doing is taking everything away. But rather from the beginning, the patient should feel more accompanied by the whole team, by the palliative care team, and by the doctor who makes decisions in other aspects as well." [...] "I believe that more and more, patients are getting used to that, to demanding information and also to assimilating and processing the information." [SPC1 - internal medicine] |
|  |  | UK | **“I think with anything like advanced care planning, the earlier the subject is raised, you hope the smoother that [process] is, and you are not in a situation where the patient is acutely unwell and trying to have all of these conversations that are a reaction to an event. I think introducing the idea early would be helpful to prepare a patient… [but] I think it’s hard, if a patient hasn’t talked about prognosis and doesn’t want to.” [UK28 - palliative care nurse]** |
|  | Clinicians are weary of overloading patients with many different conversations at this advanced stage – varying levels of patient acceptance; this stepwise process means ATT decisions can be pushed back and end up being the last medications being reviewed – as such, there remains a narrow window of opportunity to engage in shared decisions – ATT is just stopped | DK | !There’s a lot of variation, but when the patient can’t really accept where they are in their illness, it can be very difficult to have these conversations — about how this medication might no longer be beneficial — especially if it’s something they’ve been used to taking for years… and sometimes, it’s actually not the patient, but the family who can’t accept it. And if the patient lets the decision rest with their family, then that makes it hard too.” [ID7 – general practitioner] |
|  |  | FR | **“In palliative care you have to proceed in stages, because there are other things to deal with than stopping antithrombotics… imposing too many things on people is counter-productive, and that's often what you see in these antithrombotic cases: it happens very late in the patient's care, because there are things that need to be managed beforehand.” [FRC5 – palliative care]** |
|  |  | SP | “If continuing the antiplatelet or anticoagulant treatment doesn't pose much risk and helps prevent complications, we should continue with it. However, when we observe clear deterioration in the patient, and it seems they may enter the final days within a few weeks, we need to start reducing medication and stop the ones that are not necessary or do not provide any benefit. I believe there is an issue of individualization." [SPC24 - palliative nurse] |
|  |  | UK | “Yeah, I mean, some of the palliative, you know, the end, the, the true like last week or two of life I mean, we don't, you know, if that's the case, we don't discuss with the patient in a shared decision making, do you want us to stop your aspirin, we just do it.” [UK01 - vascular] |
|  | Uncertainty surrounding prognosis also further complicated timing around ATT management | DK | "I think it becomes relevant when you’re at the point where you’re tired of taking medication, or when you’re experiencing side effects and need to weigh the risks of one option versus the other. Because the actual time left — for one, we’re really not that good at predicting life expectancy. And secondly, I think it would also be difficult for the patient to suddenly have to reflect on their anticoagulant treatment when they feel the same as always. It’s been working fine. So why should I suddenly stop taking it… I think the real point to reflect on it is actually at the time of initiating the treatment — that’s when it matters." [DKC3 - oncologist] |
|  |  | FR | When I really don't expect any benefit from it, the discomfort is an obvious curse. All right, it can happen that people ask to stop, and I say yes, if you don't feel like continuing, you can probably stop. I don't like the idea of a year or life expectancy, because a year is a very long time, and when your prognosis is vital, let's say less than 3 months, it's probable, but who can say? nobody can know. One year seems too long to me to stop anticoagulant or APA treatment. [FRC1 - vascular] |
|  |  | SP | Identifying the end of life itself, this must be easier in oncology, but in cardiology it is very complicated. Because patients get worse, they could die but they don't, they go away, they seem to be fine but they stay a little worse. And with one of these [EPISODES] comes the end of life. The patient can be palliative and live two years or die in 5 days. in other words, it is very difficult to identify the end of life. [SPC10 - cardiologist] |
|  |  | UK | **“The difficulty, from my perspective is it’s often unclear that they are in the last year of life, and there have been occasions when I’ve asked oncologists can you clarify what the prognosis here is, and they’re not usually very helpful or explicit in answering that question. Now I understand it's not an exact science, but even we like, just to have an indication, they’re often, they don’t appear to be very willing to do that.” [UK18 - cardiologist]** |
|  | Triggers instead of timepoints are used instead | DK | **"If there's some kind of problem — side effects, bleeding, intolerance — then that’s often the point where we have the conversation with the patient: 'What should we do here?'" [DKC2 – cardiologist}** |
|  |  | FR |  |
|  |  | SP | “It's always the risk-benefit calculation, isn't it? And this always leads us to dilemmas, because what is more lawful is benefit. Those who have been anticoagulated for a long time find it very difficult to withdraw the anticoagulation, because it is like something they know is very important to them, and it's hard to take it back if you don't have a clear argument. Unless they bleed, then it's clear. [SPC20 – geriatrician] |
|  |  | UK | **So, I mean, in a practical setting from us, I guess when we have stopped all active treatment, that is probably a good time for us to start rationalising, because we have come to a point now where we are moving from active oncological treatment, to a more best supportive care scenario, and I think that tends to go hand in hand. Now that can happen at different time points for different patients. [UKC8 – oncologist]** |

**Theme 2: Culture of continuation**

**Sub-theme 2.1:**

| **Sub-theme** | **Essence point** | **Country** | **Quotes** |
| --- | --- | --- | --- |
| Limitations in evidence and knowledge | Limited/gaps in evidence, tools and guidance | DK | "I find it a bit difficult because you also know that cancer patients may have an increased risk of clots, so when is it one thing and when is it the other? And what's worse? Of course, it's not pleasant to have a bleed, and it can be quite severe as well. But I think maybe I needed a bit more guidance, you know, about that, really. What should weigh heavier in some way?“ [DKC5 – General Practitioner] |
|  |  | FR | **“The pendulum swings back and forth in the field of thrombosis and cancer: people are treated for the long term, and when there's no more cancer, we stop. But for people who are going to die of cancer, I don't think it's yet set in stone to systematically assess the benefit of treatment in terms of the patient's quality of life, life expectancy, generative status and tolerance.” [FRC16 - pneumologist]** |
|  |  | SP | "The challenge we have with these patients is mainly deciding the duration of the treatment, which is the cornerstone, I think it's the million-dollar question. Although there is increasingly more data suggesting that they should be treated for a very long time, as long as the cancer is active. And we know that, although the data is limited, we have more evidence that recurrences are very high if we stop the treatment. But of course, the million-dollar question is: which drug is the best? And what dose is the best? And that, indeed, takes up our time, and when it comes to deciding or discussing it with the patient, it is an important part because we need to explain or make it clear that what we are trying to do here is to prevent a new thromboembolic event. Trying to ensure that the patient has the lowest possible risk of hemorrhage.” [SPC9 - pneumologist] |
|  |  | UK | “None of the risk scores that we have for assessing bleed risk in cancer or noncancer patients are great at identifying patients. They're very clunky, identifying a tumour in situ or a patient who's elderly or anaemic. They're very basic, and the risk scores... really didn't discriminate very well from patients... So, um, yeah, I think it's really difficult, I mean, we do, we do need better risk assessment tools, um, but as with any general tool, it's very hard to apply it specifically to patients who, particularly cancer patients, who, you know, they were extremely heterogeneous in terms of their sort of comorbidity, their demographics [UK2 - haematologist] |
|  | Varied knowledge and understanding of the guidelines among clinicians / decisions not made with much thought | DK | “So there's the whole interaction problem. Before that, there was all that stuff, depending of course on what they’re receiving as well. Then there's the fact that some of the treatments they get may cause thrombocytopenia, anemia, impaired kidney function, or they’re admitted with sepsis or something like that, which ends up meaning they need to pause treatment. And then we have to keep a close eye the next time. So there are really a lot of factors that don’t come into play with, how should I put it, normal patients — non-cancer patients.” [DKC2 – cardiologist] |
|  |  | FR | “They are patients who bleed more than others because they are dehydrated, they eat less, they are cachectic. The cancer is so much there, they’re obviously not doing so well. the consequence of that is that when everything malfunctions, there is spontaneously a lot of bleeding.” [FR15 – vascular] |
|  |  | SP | “I wouldn't make any distinctions [regarding ATT deprescription] based on the type of cancer. What I sometimes make me hesitate, let's say if a patient who has had two PEs, who is anticoagulated for life, an at the end-of-life, 3-6 months away, ... let's withdraw this. Sure, maybe I would consult with the specialist. With the specialist who takes the patient, with the oncologist or even with the home palliative care team colleagues, who have more experience, to finally decide whether or not to withdraw it.” [SPC14 - GP] |
|  |  | UK | **“There's a lack of understanding, verging on ignorance, on the part of healthcare professionals who are used to managing non cancer patients, or if they’re used to managing cancer patients, they’re used to managing cancer patients in a very protocolized way… with cancer associated thrombosis, it's far more nuanced...” [UK02 - haematologist]** |
|  | Difficulty gaps in evidence poses when explaining decisions to patients, patients preferences | DK | "[Patients need to] feel reasonably well informed. And that’s extremely difficult when you can’t put exact numbers on anything." [DK1 – cardiologist] |
|  |  | FR | **“If we had more evidence based medicine, certainty about the risks, we would be more comfortable discussing it with the patient... sometimes you don’t have the answer. So it’s difficult, when you don’t have the answer to involve the patient in it.” [FRC2 - oncology]** |
|  |  | SP | ''There is a risk associated with both maintaining and withdrawing anticoagulation, it is important to provide balanced information and involve the patient in the decision.''[SPC1 - internal medicine] |
|  |  | UK | “The challenge being often I don't know the actual figures, no, it's more about sort of weighing up say, ‘seesaw’ and saying to them look, I think we're sort of moving more in this direction now where it's a bigger risk to you than it is benefit, and that's based upon those bits of history of what their bleeding is, what tools I have in the box that potentially can, prevent further bleeding.” [UK05 - oncologist] |
|  | Clinicians want/ express the need for more support, more evidence – demonstrating a reasonable decision | DK |  |
|  |  | FR | “An algorithm is always something that helps enormously in the decision-making process, not only for each member of the team, but also for patients: having clear criteria in this context, where it's very difficult, it's a bit pretentious to say that we want clear criteria, but it's the whole need for patient education that plays a role in this final decision. we also need to consider all the risks involved, and understand why we're thinking about treatment.” [FRC4 - palliative care] |
|  |  | SP | ''sometimes you feel quite alone in this decision-making [...] It would be interesting to have some tool that could help us in decision-making, yes. '' [SPC1 - internal medicine] |
|  |  | UK | **“We do just find it really difficult compared to other medications. So, somebody will be on ten meds. They’re all stopped but they’re left on their DOAC 99% of the time. I think we’d be really pleased to have some sort of guidance on it.” [UK34 – General Practitioner]** |

**Sub-theme 2.2:**

| **Sub-theme** | **Essence point** | **Country** | **Quotes** |
| --- | --- | --- | --- |
| Consequences of ATT decisions – high stakes, high uncertainty | The decision is a significant one, with competing risks either side | DK |  |
|  |  | FR | **“Unfortunately, we often have this kind of puzzle in oncology: if they bleed, we stop the anticoagulants or antiplatelets, but if we stop, they will be symptomatic on their pulmonary embolism.” [FRC2 - oncology]** |
|  |  | SP | "Sometimes I don't feel comfortable, because you know that with these patients any of the options you choose can carry complications.” [SPC1 - internal medicine] |
|  |  | UK | “So medically what would be controversial, medically it would be someone who I think has, probably both a high thrombosis risk and high bleeding risk, would be what would medically make it a difficult decision. I think what you often have to navigate in real life is the understanding that if you make a proactive decision, and there is a negative outcome, even if that is an entirely predictable negative outcome and, and you believe that that was a reasonable risk to take, erm, getting a feeling of people’s families are on board with that, is crucial, because unfortunately I think we’ve probably all had the experience where being entirely correct doesn’t shield you from the stress of attending to complaints, essentially. Erm, and sometimes it could easily feel like the safest decision is no decision, or the safest decision is to make it someone else’s decision.” [UK12 - geriatrician] |
|  | Concern about stopping over continuing | DK | "Yes, I definitely think that’s something we should be asking about. It’s both within the realm of medical defensiveness, where you’re trying to protect yourself. Because I know very well that you can easily write, ‘this medication no longer makes sense,’ but you always have to consider that the patient might then experience the very event you were trying to prevent—and someone could end up complaining about your decision. I’m aware of that. Some people might say, ‘It doesn’t matter,’ but that’s why it’s so important to involve the patient in the decision. And if the patient isn’t fully capable, then you can meet with the family and the patient to talk about it. I really think it’s important to include the patient in the decision-making process."  But just the fact that it’s written in the medical record—and I’ve become a bit more focused on the importance of shared decision-making with the patient. But it has to be documented in the record, because I know many of the lawyers at the Danish Patient Complaint Board, where I’ve also worked as a medical consultant, and they think in legal terms. They don’t think about ethics, aesthetics, or morals. It’s more about... also for the patients who don’t... So that’s why it has to be in the record.” [DKC10 – cardiologist] |
|  |  | FR | “I'd find it hard to stop: for the end of the patient who is still able-bodied and autonomous, with a high thrombo-embolic risk of stroke, because it would spoil the end of his life.” [FRC5 - palliative] |
|  |  | SP | **“We are mainly focused on symptomatic control… ATT deprescription is more difficult for us. We worry about the risk if we withdraw it [ATT]." [SPC20 – geriatrician]** |
|  |  | UK | “I would have said well, um, you've, so there's patients, not just got fibrillation, but they have, have had hard embolic events. So their, their threshold, their, their reason for having it, it's quite, it's quite high, and you’re pro-thrombotic with cancer so I would be concerned that you stop it and they are gonna have there's, there's a higher risk of, of a life ending or, embolic event.” [UK01 - vascular] |
|  | ATT risk is more acceptable than the risk of stopping, including the repercussions legally and from the family | DK | “I documented that in the record—because you never know. There could be a situation where the patient suddenly has a stroke, and then the family thinks it’s absolutely terrible that, on top of everything, their father had to suffer a stroke in his final days.” [DKC10 – cardiologist] |
|  |  | FR | I've become part of the norm: do it like that. as soon as you do it differently, you have to justify it.” [FRC5 – palliative care] |
|  |  | SP | "In an advanced metastatic or non-curative context, it's true that unless there is a hemorrhagic problem or a clear contraindication that puts the patient at risk, we do not stop anticoagulation.” [SPC8 - oncologist] |
|  |  | UK | **“I think what you often have to navigate in real life is the understanding that if you make a proactive decision, and there is a negative outcome, even if that is an entirely predictable negative outcome and you believe that was a reasonable risk to take… I think we’ve probably all had the experience where being entirely correct doesn’t shield you from the stress of attending to complaints and sometimes it could easily feel like the safest decision is no decision, [or] to make it someone else’s decision.” [UK12 - geriatrician]** |
|  | Hesitance to bring up ATT review with patients who feel strongly about their ATT medication – the consequences and burden on the patient | DK | **“If they have it in their mind that this is very important and they’re strongly convinced that it’s life-saving medication, then I don’t think I would correct them at this stage — not if I don’t believe it myself. I mean, assuming there aren’t any other red flags.” [DKC1 – cardiologist]** |
|  |  | FR | “For me, either a reflection comes from the patient and we leave the door open and that’s important: we are here to leave them (anticoagulants) as long as you ask yourself the question to discuss it seems important to me. On the other hand, there is a risk of increased tension to come proactively to be able to stop if there has been no request from the patient, no change in the intake, no bleeding event.” [FRC10 - home hospitalisation] |
|  |  | SP | ''It would be very helpful if the doctor who attends to the patient is always the same throughout the process, [...] if it were the same doctor who prescribed the medication who undertakes the deprescribing, and the patient would then have trust in that doctor's judgment.'' [SPC1 - internal medicine] |
|  |  | UK | “I think the patients that, have been on things for a long time are less comfortable about you stopping things. Erm, because if they’ve had recurrent DVTs, I don’t know, 20 - 30 years ago and they have been on anticoagulation since and now you are stopping something that has kept me, you know, in their head, you know alive for 20 years that is a more difficult conversation. I think, if it’s treatment related and it’s small and they’ve not been symptomatic and we are saying look, we picked this up on your scan, it probably relates to your cancer and the treatment, we want to give you some injections or something, and then some tablets to try and stop it coming back, I think that is a more comfortable decision for patients because it's, it’s newer, they’ve been less bothered by it, you know, they almost see it as part of their sort of cancer treatment as a whole, and it comes in a package with that so they are more comfortable. But I find it’s the patients who have been on medication for a long time that are less comfortable with stopping things.” [UKC8 – oncologist] |

**Sub-theme 2.3:**

| **Sub-theme** | **Essence point** | **Country** | **Quotes** |
| --- | --- | --- | --- |
| Passivity in ongoing ATT prescription – a deep routed practice | Concept of stopping – not present in wider culture / compared to other medications | DK | “There’s this group of medications that we’re maybe not exactly unconcerned about, but that we’re less inclined to address — or not as comfortable with, you could say. That includes, for example, erectile dysfunction medications. We generally prefer not to say much about those. I do bring it up sometimes, though — because I actually think it’s something we should talk about now and then. But it definitely falls into that category… not quite the ‘naughty corner,’ but erectile dysfunction meds are much more in the naughty corner than Fragmin is. [DKC15 – palliative care nurse] |
|  |  | FR |  |
|  |  | SP | “I deprescribe not as much as it represents that it should be done, but... of course, deprescribing is like very important, but it's hard for doctors. It is easier to add drugs than to remove them. I tend to describe quite a lot, especially when they are admitted. For those who are outpatients it is less common to deprescribe.” [SPC10 - cardiologist] |
|  |  | UK | “I think it's an easy drug to start, but so are lots of medicines, you know, most doctors are comfortable starting medicines, it's harder to stop medicines.” [UK03 - palliative care] |
|  | ATT in particular is difficult to consider stopping – some are more comfortable, but sense many others are not | DK | "No, I think it’s probably the first one—that they’re started on it, and then it just continues. And I don’t think anyone, myself included, really steps in. Because I wouldn’t even know if stopping it is the right thing to do, you know? So that’s what I think.” [DKC9 – vascular surgeon] |
|  |  | FR | **“It makes everyone else uncomfortable, that's for sure, because it's a dogma to leave it until the end… everyone continues as they have done for years, you're the only one to do (to stop), I've become part of the norm: do it like that. as soon as you do it differently, you have to justify it.” [FRC5 – palliative care]** |
|  |  | SP | “They are [PALLIATIVE CARE] patients that you have to constantly review the medication, and assess whether with that context the medicines they are taking are worth it, if they are still indicated. Of course, this is usually not done as regularly as it should be done. This is indeed considered in the last days of life, and then also sometimes before. [PROBABLY] Even before this [DEPRESCRIPTION] should be done. Of course, maybe you say no, no, let's wait later to finish withdrawing it. You will probably withdraw sooner the statin than the antiplatelet or the anticoagulant. You'll keep this [ATT] longer, until a time comes when you say it's not worth it, let's take that out too.” [SPC14 - GP] |
|  |  | UK | **“There is always the difficulty about stopping them. If somebody’s had a clot in everyday life, we get a nice, neat letter from Haematology saying, ‘Take for three months, stop it’ and everybody’s really happy with that. But the patients who are on anticoagulation, for cancer, it is really difficult. It is the stopping of it, that, that trips us up really. And it’s probably the last medication to be stopped in lots of cases.” [UK34 - GP]** |
|  | Alternatives / an effort to stop / continuation is easier | DK | I think in most cases, the continuation of treatment is a passive decision. Not always, but mostly. And I think some people lose track of what kind of thrombosis we’re actually dealing with — like with peripheral pulmonary emboli. I really think some clinicians get lost in figuring out which types of clots and risks genuinely warrant anticoagulant treatment and which don’t. And then they prefer to be on the safe side — because everyone’s heard that a clot is dangerous, so we should treat it. And you also feel like you've done something — you've prescribed something that day. But yes, I think for the most part, it’s passive." [DKC13 – palliative care] |
|  |  | FR | “[take into account] what the other doctors who follow the patient think. If the oncologist is rather favourable to maintain it [ATT], I will not fight, I understand, and this is the plurality of medical views in this situation. That’s the whole point, that continuation is an easy decision.” [FRC10 - home hospitalisation clinician] |
|  |  | SP | "If there is obviously significant bleeding or significant thrombocytopenia, you always ask, assess, request a consultation... sometimes you don't deprescribe but reduce the dose, you always evaluate the risk-benefit, which is sometimes not so easy in clinical practice.” [SPC13 - GP] |
|  |  | UK | **“There’s more of an effort to stop them in a way, because you have to sort of really talk to the patient about it… whereas it’s easier to maintain the status quo, of nice and safe, we’re on anticoagulation… let’s not rock the boat.” [UK06 - palliative care]** |
|  | There is a lack of overt triggers to ATT review | DK |  |
|  |  | FR | “I don’t change anything unless he bleeds or has trouble eating. I am not active in the stop if there is no bleeding or no deglobulization so in the absence of complication I continue.” [FRC10 - home hospitalisation] |
|  |  | SP | "In an advanced metastatic or non-curative context, it's true that unless there is a hemorrhagic problem or a clear contraindication that puts the patient at risk, we do not stop anticoagulation. [...] If the patient has a very short life expectancy, then it's a bit... but if the patient is not bothered by it and it doesn't cause problems, we continue with anticoagulation because there is a risk-benefit, the risk of pulmonary thromboembolism, deep vein thrombosis... or if they have something that could worsen. But if it's a patient who is at the end of life or it causes discomfort or the palliative care team doesn't consider it indicated, I think it should be stopped.” [SPC8 – oncologist] |
|  |  | UK | **“We're without a trigger now, so I think unless there is some sort of trigger where people say, okay, the surprise question, or anticipatory medications, or, something to make the trigger of ‘should we continue’?” [UK03 - palliative care]** |
|  | Majority see it as a passive decision | DK | "Most of the time, it’s a passive decision—they simply continue without anyone intervening. But then there are also a few patients who actively make that decision themselves. I come across that from time to time.” [DKC8 – oncologist] |
|  |  | FR | “When the risk of bleeding is negligible and treatment is really necessary and there is a risk of having more adverse effects to stop it, I am in favour of continuing it until the end. because if there is no risk of bleeding found on a given day, I do not see why we would deprive the patient of effective treatment. It would be good if we were active and that, at any time of the takeover, we regularly ask ourselves the question of the risk benefit balance, and that we give ourselves the right to stop. But I recognize that right now it’s pretty passive, that is to say, we let things run, and then we wait a little while for events to happen. maybe it’s a little too late.” [FRC12 - geriatrician] |
|  |  | SP | “I think it's often by default. I mean, reviewing... I mean, stopping, reviewing all the medications, and deciding 'this one yes, this one no,' that's a huge task! And on top of that, explaining to the patient, that's a job that requires sitting down...It's a big job.” [SPC24 - palliative nurse] |
|  |  | UK | “It's the meds just continue, you know it's, it's that masterly inactive, well, not masterly activity, it's inactivity. There are, there are no triggers, um, unless somebody bleeds. So it's when somebody bleeds, renal function goes off, people suddenly think, oh my gosh, right, what's happened? What meds have we got going? But otherwise it's, the things just carry on.” [UK03 - palliative care] |
|  | Sense that ATT goes unnoticed | DK | “I don’t think that happens. So it really depends on the individual—whether someone notices it. Like, does one of our nurses notice it? Does the palliative care doctor notice it and then ask, 'Does this still make sense?' So that’s why I think—if that happens—then of course it becomes a conscious decision. You could say, 'Should we continue or should we stop?' and then it’s a deliberate choice. I’m only involved in the cases where it becomes a conscious decision, but it may be that there are many cases where things just keep going. Because you think—well, right now there are so many other priorities, so you just let the medication continue.” [DKC2 – cardiologist] |
|  |  | FR |  |
|  |  | SP | **"I believe that the issue of anticoagulation is something that is undervalued, undertreated, and not given the importance it actually requires, and sometimes it even goes unnoticed… these are things that I believe we need to change, and they need to change from within, from ourselves as professionals." [SPC4 – general practitioner]** |
|  |  | UK | “No I don’t, and something I probably should do but I guess it’s, particularly when I haven’t prescribed it in the first place then it’s not so much that it’s not my decision, but I think it’s just not so much in my head to be honest, it’s just not something that I think about. As you said when you are faced with a situation because they are having a complication from anticoagulation, that’s an easier scenario.” [UK08 - oncologist] |
|  | Distinction between ATT indication/ATT type – easier, but often overlooked | DK | **"I think aspirin is a very typical medication that often doesn’t get much attention. So there could definitely be more [cases]. And it would be one of the first things you'd consider pausing if it’s only being used for prevention." [DKC3- oncologist]** |
|  |  | FR | “To stop AC treatment, I'd stay with the idea that if we say that the end of life is to stop oncological treatments, we'll see how the patient's general condition is and how he's moving around. For the primary prevention indication of APA, without ever having stent surgery or atheromatous disease, I wouldn't ask myself the question, I'd stop.” [FRC16 – pneumologist] |
|  |  | SP | "A treatment with antiplatelet agents is easier to suspend because generally, the reasons for taking antiplatelet agents have been pre-existing and for other causes. So, when we review medications and can suspend some, we always suspend the antiplatelet agents." [SPC6 – palliative care] |
|  |  | UK | “Sometimes patients are on antiplatelets and if you dig for the indication it’s not clear and if it isn’t anything too strong I’d be more inclined to stop it, but to be honest… I would probably be quite slow at stopping anticoagulation or antiplatelets in patients with advanced malignancy.” [UKC19 – pneumologist] |
|  | Contrast – some describe being more at ease with deprescribing ATT – see it as an active decision | DK | “I experience it as an active decision—that we look at the whole, overall picture. Yes. I do think it’s an active decision.” [DKC14 – palliative care nurse] |
|  |  | FR | **“I'm at ease [with ATT deprescription]; it makes everyone else uncomfortable, that's for sure, because it's a dogma to leave it until the end.” [FRC5 – palliative care]** |
|  |  | SP | “It [ATT DEPRESCRIPTION] does not pose a great challenge to us. It's almost routine. But well, the objectives would be, one, that it does not bleed, it would be hemorrhagic complications. That is, efficacy and safety. One would be that they are well anticoagulated, but this applies especially to those with mechanical prostheses, that if you have them badly anticoagulated you do have a greater risk of thrombosis. With AF as well, but if they are poorly anticoagulated for a few days, nothing happens. Patients are increasingly frail, older, and thus, the main problem with anticoagulants is the risk of bleeding.” [SPC10 - cardiologist] |
|  |  | UK | “I don’t mind making decisions, erm, I’m generally quite, I think, as a rule, quite proactive about discontinuing medications when the patients have life limiting illnesses, erm, and generally quite enthusiastic about stopping medication because I see an awful lot of harm that comes from people being on treatment for all sorts of different things, so if it’s not needed I am quite enthusiastic about stopping treatment.” [UK19 - respiratory] |
