# Supplementary material for: Clinician perspectives on antithrombotic therapy management in advanced cancer: a multinational qualitative study
Source: Res Pract Thromb Haemost. 2026 Mar 25;10(3):103427. doi: 10.1016/j.rpth.2026.103427 (PMC13092592; doi:10.1016/j.rpth.2026.103427)
Supplement: Supplementary File 3 [file mmc3.docx]

**Supplementary File 3: Suggestions for the shared decision support tool (SDST)**

| **Suggestions for the SDST** |
| --- |
| - Palliative care and primary care clinicians (GPs) are best placed to use the tool / lead ATT decisions in the context of advanced cancer, and end of life. However, it must be noted the variability in transitions of care, differences in responsible clinicians and their own personal barriers to feeling responsible. In particular, support from ATT specialists was valued. - Clinicians need evidence that address the complexities of this decision, to support their role in ATT decision making, to assure patients of the credibility of the decision being considered, and to address risk and uncertainty. To support this, guidance/education/training to build their knowledge and confidence in this decision and communicating these decisions is needed. - However, there are cautions that need to be addressed in clinician training, including identifying the right time to introduce the decision, explaining the decision is reasonable and logical, and care in not overburdening patients with information/decisions, and sensitivity regarding end-of-life care context. - Time and resources for preparing for shared decision making (both clinician and patient/relative preparation) is essential. - Clinicians want to promote patient autonomy and make a shared decision with the patient, including understanding the individual patient context and preferences. Clinicians understand that there is variation in information needs, health literacy and cognitive ability, and advocate for this understanding as a basis prior to initiating in shared decisions. - In the absence of a specific triggering factor to initiate ATT decisions, integration with, or transition to, palliative care might be a good time to review risks and introduce the decision. There needs to be continuity of care during this integration/transition. However, this event is not necessarily a consistent event among patients, so other key transition points need to be considered. |
